# Comparative analysis of mini-open trans-thoracic transpleural and posterior approaches in thoracic disc herniation surgery: A 10-year retrospective review

**DOI:** 10.1016/j.bas.2025.104244

**Published:** 2025-03-28

**Authors:** Ali Baram, Giorgio Cracchiolo, Marco Riva, Gabriele Capo, Leonardo Anselmi, Carlo Brembilla, Stefania Radice, Maria Pia Tropeano, Carla Anania, Emanuela Morenghi, Maurizio Fornari, Federico Pessina

**Affiliations:** a Department of Neurosurgery, IRCCS Humanitas Research Hospital, Via Alessandro Manzoni 56, 20089 Rozzano, Milan, Italy; b Department of Biomedical Sciences, Humanitas University, via Rita Levi Montalcini 4, 20072 Pieve Emanuele, Milan, Italy; c School of Medicine and Surgery, University of Milano-Bicocca, Bergamo, Italy; d Biostatistics Unit, IRCCS Humanitas Research Hospital, via Manzoni 56, 20089 Rozzano, Milan, Italy

**Keywords:** Thoracic disc herniation, Thoracic myelopathy, Surgical treatment, Anterior approach, Posterior approach, Spinal surgery

## Abstract

**Introduction:**

Thoracic disc herniations (TDHs) are rare, and surgery is typically reserved for patients with radiculopathy, myelopathy, or intractable back pain. Despite established algorithms, the optimal surgical strategy remains debated.

**Research question:**

What are the clinical, surgical, and radiological outcomes of anterior and posterior surgical approaches for TDHs over a 10-year period?

**Material and methods:**

A retrospective analysis of 54 patients who underwent surgery for TDHs (2013–2022) was performed. Patients were grouped by surgical approach: anterior (34 patients) and posterior (20 patients). Data included preoperative and postoperative outcomes such as operative times, hospital stays, complications, reoperations, and assessments using the Frankel scale, Nurick score, and Visual Analog Scale (VAS) for pain.

**Results:**

Both approaches improved clinical outcomes. No significant differences in postoperative Nurick or VAS pain scores were observed. However, the anterior approach showed better Frankel score improvements but was associated with longer operative times and hospital stays. Complications were more frequent in the anterior group.

**Discussion and conclusion:**

Both approaches effectively alleviate symptoms in symptomatic TDHs. The anterior approach offers greater neurological improvement but carries higher complication risks. Surgical strategy should be tailored based on herniation characteristics and surgeon expertise. Anterior approaches are ideal for central, large, or calcified herniations, while posterior approaches are preferable for lateral ones.

## Highlights

• Anterior approaches improve neurological outcomes but increase operative times and hospital stays.
• Anterior approaches are preferred for complex, giant, calcified thoracic herniations.
• Posterior approaches are used for lateral, soft thoracic herniations with fewer complications.
• Treatment tailored to herniation characteristics optimizes surgical outcomes.

## 1 Introduction

**Fig. 1:**
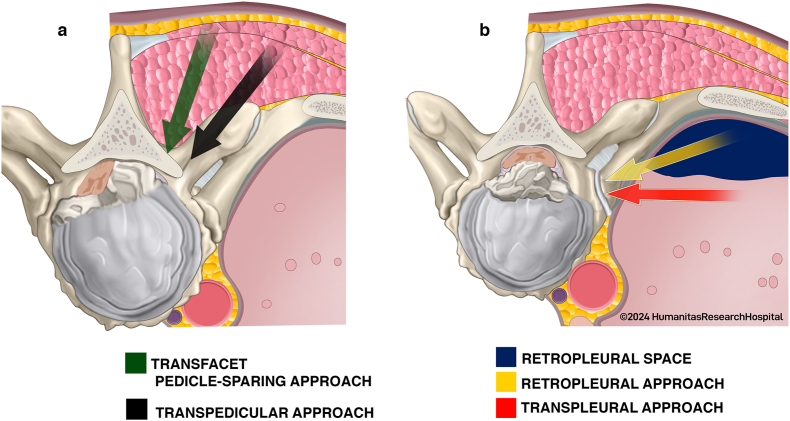
Surgical Approaches for Thoracic Disc Herniation Removal. (A) Posterior approaches include the transfacet pedicle-sparing approach (green arrow) and the transpedicular approach (black arrow). (B) Anterior approaches include the retropleural approach (yellow arrow) and the transpleural approach (red arrow). The retropleural space (blue) is highlighted to illustrate the anatomical corridor used during the retropleural approach.

Thoracic disc herniations (TDHs) are a relatively rare pathology within the general population, constituting 0.1 %–3 % of all disc herniations (Carson et al., 1971; Arce and Dohrmann, 1985). Predominantly located in the lower thoracic spine (T8-T12), TDHs vary in size from small to giant, in consistency from soft to calcified, and in position from central to paracentral, ultimately resulting in the anterior compression of nervous structures (Wood et al., 1995). Accounting for less than 1 % of all disc surgeries, TDH surgery is typically reserved for patients with severe or progressive myelopathy, persistent axial back pain, and/or intractable radiculopathy (Court et al., 2018). In 1934, Mixter and Barr introduced decompressive laminectomy as the first reported surgical approach for the treatment of TDHs (Mixter and Barr, 1964). However, this approach was later discarded due to significant morbidity and mortality rates. From the 1960s onwards, a spectrum of alternative surgical approaches emerged. Posterior routes (transpedicular, transfacet pedicle-sparing, costotransversectomy, and lateral extracavitary approaches) are more familiar to spine surgeons, but necessitate more extensive soft tissue dissection and allow poor direct herniation visualization. Conversely, anterior routes (transthoracic transpleural or retropleural, mini-open retropleural, thoracoscopic, and full-endoscopic) provide direct visualization of the herniation, enhancing resection, and often obviate the need for spinal fixation, albeit demanding a steeper learning curve (See Fig. 1). (Yoshihara, 2014) Both approaches report overall reoperation and morbidity rates as high as 5.9 % and 29 %, emphasizing the importance of tailoring treatment to minimize complications (Brotis et al., 2019; Uribe et al., 2012). Despite previous efforts to define treatment algorithms, the appropriate surgical strategy for TDHs remain controversial. In an effort to better delineate surgical morbidity, functional outcomes, and potential preoperative criteria guiding the choice between anterior and posterior approaches, we conducted a retrospective analysis of consecutive patients treated for symptomatic TDHs in our institution over a decade.

## 2 Materials and methods

### 2.1 Study design and patient population

We included patients who underwent surgical intervention for TDHs at our institution between January 2013 and December 2022. The patient cohort was stratified into three groups: those who underwent an anterior approach, those managed through a posterior approach, and individuals with a history of unsuccessful TDH surgery elsewhere. To ensure results homogeneity, patients undergoing reoperation at our institution (third group) were described as a separate group and not included in the main statistical analysis. Follow-up assessments were conducted at the last follow-up, primarily in an outpatient setting by two of the authors, LA and SR. When an in-person evaluation was not possible, telematic interviews were conducted, accounting for approximately 5 % of the assessments. For follow-up evaluations, we used a standardized evaluation sheet, which consisted of handwritten notes in which clinical and functional outcomes were recorded. The term 'standardized' refers to the consistent use of validated clinical scales and questionnaires, as detailed in the methods section, rather than a structured, pre-printed document. This ensured uniformity in data collection across all patients, including those assessed telematically. Even in the small proportion of patients assessed remotely, improvement was evident and well-documented, and all had undergone prior in-person assessments confirming their clinical progression over time. The study received approval from the local ethics committee (approval number 1575473) and adhered to the principles of the 1964 Declaration of Helsinki and its amendments.

### 2.2 Radiographic imaging

Preoperative magnetic resonance imaging (MRI) and computed tomography (CT) scans were systematically performed for all patients to document the lesion's level, axial localization (central or paracentral), size (giant or non-giant) and consistency (soft, calcified, or a combination of both). Giant TDHs were defined as those occupying at least 40 % of the spinal canal's diameter (Hott et al., 2005). Furthermore, we manually calculated the residual spinal canal area (rSCA), which corresponds to the canal area not occupied by the TDH, expressing it in both mm^2^ and as a percentage of the total spinal canal area (tSCA). The Delta rSCA indicate the postoperative rSCA minus the preoperative rSCA. The spinal canal area was manually calculated using the measurement function available on the PACS viewer program routinely used for medical imaging review. The calculation was performed by selecting multiple points to outline the area of interest, after which the PACS system automatically computed the enclosed area. No additional software was used for this measurement.

### 2.3 Data collection and outcome measures

We collected patient demographics and comorbidities. Charlson Comorbidity Index (CCI) and the American Society of Anesthesiologists (ASA) classification were used as comprehensive comorbidity indicators (Charlson et al., 1987; Doyle et al., 2023). Clinical data included disease duration, defined as the period from the onset of the patient's initial symptoms, such as paresthesias or any type of neurological deficit, to the time of surgical intervention. Additional clinical data included the presence of myelopathy and/or radiculopathy, Visual Analog Scale (VAS) pain score, neurological status scores (Frankel scale, Nurick score), and assessment of sphincter functionality (normal, incontinent, or retentive) (Nurick, 1972; Frankel et al., 1969). Patients were categorized by anterior/posterior surgical approach, and additional data included intraoperative neuromonitoring (IONM) use, neuronavigation, method of spinal stabilization (if performed), surgical time, hospital stay, complications, reoperations, and mortality.

### 2.4 Surgical indication and technique

Patients presenting with axial pain, radiculopathy, or myelopathy attributed to TDH were identified as potential candidates for surgical intervention. For those without signs of myelopathy, a mandatory three-month period of conservative nonoperative management was implemented. The choice of operative route hinged upon the size, location, and consistency of the TDH and aimed to establish a direct corridor to the TDH under visual guidance, minimizing manipulation of the spinal cord. Intraoperative neuromonitoring was employed in almost all of our cases. In cases of central TDHs, particularly when giant and/or calcified, the preferred route was the anterior approach, specifically the mini-open trans-thoracic transpleural approach. This approach directly addresses the TDH without retracting the spinal cord. The anterior approach was always performed in collaboration with a thoracic surgeon, who remained the same throughout the study period and was responsible for the surgical access. The procedures themselves were performed by two senior spine surgeons with extensive experience in both anterior and posterior approaches. Neither of the two surgeons was exclusively dedicated to a single approach, ensuring a balanced distribution of cases between them. Conversely, patients with more laterally located TDHs underwent a posterior approach, such as laminectomy or a posterolateral approach, which could be either transpedicular or transfacet pedicle-sparing. Spinal stabilization and fusion were reserved for cases where extensive bony resection might have led to iatrogenic spinal instability.

### 2.5 Statistical analysis

Given the rarity of thoracic disc herniations, we included all patients treated at our institution over a 10-year period, resulting in an estimated sample size of about 50 patients. This sample size provided sufficient power to detect significant effects only for large differences, typically with an effect size greater than 0.8. The data were presented in various formats depending on their nature: categorical data were expressed as numbers and percentages, continuous variables with an approximately Gaussian distribution were described using mean and standard deviation, while non-normally distributed continuous variables were represented by the median and range. We assessed adherence to a Gaussian distribution using the Shapiro-Wilks test. For variables showing clinically important differences, we calculated Cohen's d effect size along with the 95 % confidence interval (95 % CI). Cohen's d quantifies the magnitude of differences between two groups in standardized units, independent of sample size, and is commonly interpreted as follows: 0.2 represents a small effect, 0.5 a moderate effect, and 0.8 or greater a large effect. This approach allowed us to assess the practical relevance of differences between surgical approaches while avoiding the limitations of underpowered hypothesis testing. All statistical analyses were conducted using Stata version 18.

## 3 Results

### 3.1 Patient general characteristics

This study included 58 consecutive patients with a total of 58 symptomatic TDHs, with an average follow-up period of 70 months (range 12–118 months). Of these patients, 54 were surgery-naïve, while 4 had previously been treated elsewhere. Among the surgery-naïve patients, 34 (63 %) underwent an anterior transthoracic mini-open approach, while 20 (37 %) were treated with a posterior approach. In the posterior group, 3 patients underwent laminectomy, 4 underwent a transfacet-pedicle sparing approach, and 13 received a transpedicular approach. The cohort consisted of 31 males (57.4 %) and 23 females (42.6 %) with a mean age of 54 ± 14.8 years (range 17–81 years). The average BMI was 26.7 ± 4.2, and 13 patients (24 %) were smokers. Notably, none of the patients had major comorbidities that influenced the surgical decision-making process. Further details regarding the general characteristics of the patients are provided in Table 1.

**Table 1:** Patient demographics and comorbidities BMI, Body Mass Index; ASA, American Society of Anesthesiologists; CCI, Charlson Comorbidity Index; LOD, Length of Disease.

	Total (n = 54)	Anterior (n = 34)	Posterior (n = 20)
Age	54.0 ± 14.8	51.5 ± 14.5	58.3 ± 14.8
Sex (M)	31 (57.4 %)	21 (61.8 %)	10 (50.00 %)
Smoke	13 (24.1 %)	5 (14.7 %)	8 (40.0 %)
BMI	26.7 ± 4.2	26.9 ± 4.2	26.2 ± 4.4
ASA			
1	32 (59.3 %)	24 (70.6 %)	8 (40.00 %)
2	12 (22.2 %)	6 (17.7 %)	6 (30.00 %)
3	10 (18.5 %)	4 (11.8 %)	6 (30.00 %)
CCI	1 (0–6)	1 (0–6)	2 (0–6)
LOD (months)	7.5 (1–84)	7.5 (1–36)	8 (1–84)

### 3.2 Radiological characteristics

**Fig. 2:**
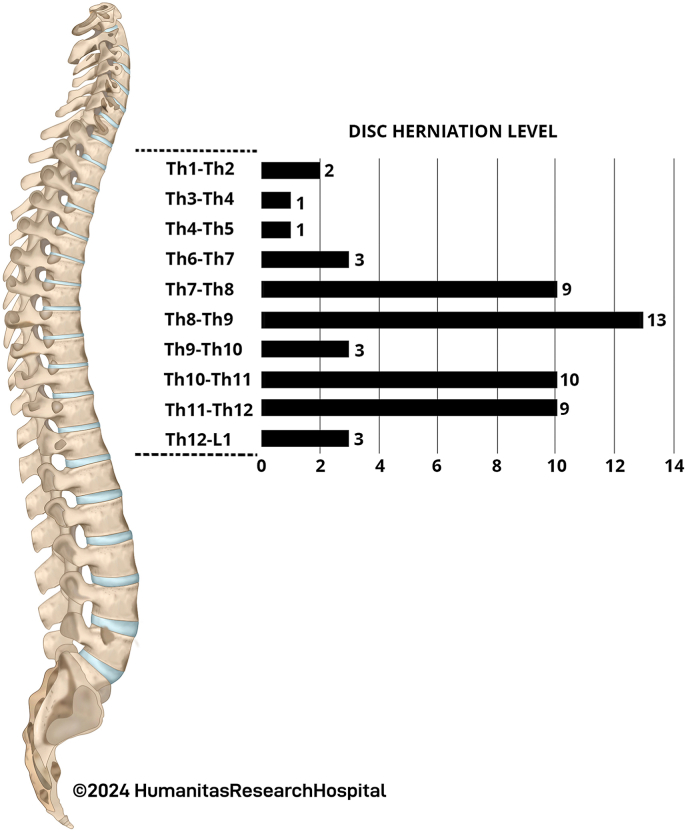
Distribution of Thoracic Disc Herniation Levels. The bar chart illustrates the distribution of thoracic disc herniations by spinal level among the patient cohort. The most common levels of herniation are T8-T9 and T10-T11, with 13 and 10 cases, respectively.

Among the 54 cases of symptomatic TDHs, 20 (37 %) were classified as giant, while 34 (63 %) were non-giant. Complete calcification was observed in 36 cases (66.7 %), with 7 cases (13 %) identified as soft and 11 cases (20.4 %) containing both soft and calcified components. The distribution included 23 (42.6 %) central and 31 (57.4 %) paracentral TDHs, with 2 out of 54 cases (3.7 %) presenting intradural extension. The distribution of TDH levels is illustrated in Fig. 2. The majority of calcified and central disc herniations were treated through an anterior approach, whereas the majority of soft or partially calcified and paracentral disc herniations were managed with a posterior approach. Preoperatively, the percentage of rSCA was 49.0 % ± 15.6, which increased to 86.7 % ± 9.8 after surgery. The Delta rSCA remained consistent between patients treated with the anterior approach (38.9 % ± 17.1) and those treated with the posterior approach (35.2 % ± 15.5). A summary of the radiological results can be found in Table 2.

**Table 2:** Thoracic disc herniations radiological features tSCA, total spinal canal area; rSCA, residual spinal canal area.

	Total (n = 54)	Anterior approach (n = 34)	Posterior approach (n = 20)
**TDH Characteristics**			
Giant	20 (37.0 %)	15 (44.1 %)	5 (25.00 %)
Type			
Calcific	36 (66.7 %)	28 (82.4 %)	8 (40.00 %)
Soft	7 (13 %)	3 (8.8 %)	4 (20.00 %)
Both	11 (20.4 %)	3 (8.8 %)	8 (40.00 %)
Position			
Central	23 (43 %)	18 (52.9 %)	5 (25.00 %)
Paracentral	31 (57.4 %)	16 (47.1 %)	15 (75.00 %)
Intradural disc	2 (3.7 %)	2 (5.9 %)	0
**Radiological Data**			
tSCA (mm^2^)	202 ± 40	202 ± 34	204 ± 49
Preoperative rSCA (mm^2^)	100 ± 39	97 ± 36	106 ± 43
Postoperative rSCA (mm^2^)	176 ± 42	175 ± 40	178 ± 46
Delta rSCA (mm^2^)	76.2 ± 36.7	77.7 ± 36.3	73.3 ± 38.6
Preoperative rSCA (%)	49.0 ± 15.6	47.6 ± 14.0	51.4 ± 15.6
Postoperative rSCA (%)	86.7 ± 9.8	86.5 ± 10.9	87.1 ± 7.4
Delta rSCA (%)	37.6 ± 16.5	38.9 ± 17.1	35.2 ± 15.5

### 3.3 Clinical outcome

Of the entire cohort, 23 patients presented with myelopathy alone, 12 experienced only radicular symptoms, and 19 exhibited both. The preoperative VAS had a median of 4.5 (range 0–9), showing improvement post-surgery (median of 0, range 0–5). However, 9 patients (16.7 %), all of whom underwent anterior approaches, reported persistent thoracic pain. Regarding neurological function, 11 patients (20.4 %) had a Frankel score of C before surgery, 29 scored D (53.7 %), and 14 scored E (25.9 %). After surgery, 3 patients (5.6 %) showed a Frankel score of C, 17 scored D (31.5 %), and 34 scored E (63 %). Overall, 24 patients (44.4 %) showed improvement in their Frankel score, while 30 patients (55.6 %) remained stable, and none experienced worsening. The Nurick score exhibited a preoperative median of 2 (range 0–5) and improved to a median of 0 (range 0–5) post-surgery, with a delta of −1.42 ± 1.13. Before surgery, sphincter function was normal in 38 patients (70.4 %), while 11 patients (20.4 %) exhibited incontinence, and 5 patients (9.3 %) showed retention. Post-surgery, there was notable improvement, with only 3 patients (5.6 %) experiencing persistent incontinence (see Table 4).

**Table 3:** Surgical Features IO, intra-operative.

	Total (n = 54)	Anterior approach (n = 34)	Posterior approach (n = 20)
Surgical time (min)	172 ± 68	195 ± 56	134 ± 70
Fixation	10 (18.5 %)	2 (5.9 %)	8 (40.00 %)
IO Neuromonitoring	23 (42.6 %)	18 (52.9 %)	5 (25.00 %)
IO Navigation	50 (92.6 %)	31 (91.2 %)	19 (95.00 %)
Length of stay (days)	8.5 (3–54)	11 (5–54)	5 (3–15)
Complications	10 (18.5 %)	7 (20.6 %)	3 (15.00 %)
Reoperations	5 (9.3 %)	3 (8.8 %)	2 (10.00 %)
Death	3 (5.6 %)	3 (8.8 %)	0

**Table 4:** Clinical outcomes.

	Total (n = 54)	Anterior approach (n = 34)	Posterior approach (n = 20)
**Preoperative symptoms**			
Radiculopathy only	12 (22.2 %)	8 (23.5 %)	4 (20.00 %)
Myelopathy only	23 (42.6 %)	18 (52.9 %)	5 (25.00 %)
Both	19 (35.2 %)	8 (23.5 %)	11 (55.00 %)
**VAS**			
Preoperative VAS	3.7 ± 3.2 4.5 (0–9)	2.7 ± 3 1 (0–9)	5.4 ± 2.8 6 (0–9)
Postoperative VAS	0.9 ± 1.3 0 (0–5)	0.6 ± 0.9 0 (0–3)	1.3 ± 1.8 0 (0–5)
Delta VAS	−2.8 ± 3.0	−2.0 ± 2.9	−4.2 ± 2.9
**Frankel**			
Preoperative Frankel			
C	11 (20.4 %)	6 (17.7 %)	5 (25.00 %)
D	29 (53.7 %)	19 (55.6 %)	10 (50.00 %)
E	14 (25.9 %)	9 (29.5 %)	5 (25.00 %)
Postoperative Frankel			
C	3 (5.6 %)	1 (2.9 %)	2 (10.00 %)
D	17 (31.5 %)	10 (29.4 %)	7 (35.00 %)
E	34 (63 %)	23 (67.7 %)	11 (55.00 %)
Improved Frankel	24 (44.4 %)	16 (47.1 %)	8 (40.00 %)
Stable Frankel	30 (55.6 %)	18 (52.9 %)	12 (60.00 %)
**Nurick**			
Preoperative Nurick	2 (0–5)	2 (0–5)	2 (0–5)
Postoperative Nurick	0 (0–5)	0 (0–5)	0 (0–5)
Delta Nurick	−1.4 ± 1.1	−1.6 ± 1.2	−1.2 ± 1.0
**Sphincters functionality**			
Preoperative function			
Normal	38 (70.4 %)	20 (58.8 %)	18 (90.00 %)
Incontinence	11 (20.4 %)	10 (29.4 %)	1 (5.00 %)
Retention	5 (9.3 %)	4 (11.8 %)	1 (5.00 %)
Postoperative function			
Normal	51 (94.4 %)	32 (94.1 %)	19 (95.00 %)
Incontinence	3 (5.6 %)	2 (5.9 %)	1 (5.00 %)
Retention	0	0	0

VAS, Visual Analog Scale; NRS, Numerical Rating Scale.

### 3.4 Surgical findings and postoperative course

The average duration of operative procedures was 172 ± 68 min, with a notable difference between the anterior (195 ± 56 min) and posterior approaches (134 ± 70 min), resulting in an effect size of −1.00 (95 % CI: 1.58 to −0.41). Anterior plate and screw placement was performed in two patients (5.9 %) within the anterior approach group, while posterior screw and rod fixation was performed in eight patients (40 %) in the posterior group. Intraoperative neuromonitoring was employed in 18 out of 34 cases (53 %) for anterior approaches and in 5 out of 20 cases (25 %) for posterior approaches, as its routine use began only after 2017 in our unit. Similarly, intraoperative neuronavigation was applied in 92 % of cases following its introduction in 2014. The median length of hospital stay was 8.5 days (range 3–54), with the anterior approach group exhibiting a median stay of 11 days (range 5–54) and the posterior approach group a median of 5 days (range 3–15), with a Cohen's d effect size of −0.91 (95 % CI: 1.49 to 0.33). Complications occurred in 10 patients (18.5 % of the total), with an overall reoperation rate of 9.3 % (5/54) (see Table 3). In the anterior approach group, complications included three cases of pleural effusion, one pneumothorax, and one postoperative iatrogenic vertebral fracture that required posterior fixation surgery. Additionaly, two cases had incomplete resection with residual compressive TDH, necessitating reintervention. Among patients who underwent a posterior approach, complications included one case of spondylodiscitis, which was managed pharmacologically; one wound infection requiring surgical intervention; and one cerebrospinal fluid (CSF) leak that required reoperation for dura sealing and lumbar drain placement. Three patients (5.6 %), all from the anterior approach cohort, died during follow-up. Two of these deaths were attributed to medical causes occurring a significant time after surgery, while the third was due to herpetic encephalitis occurring three months after the procedure. The third group of patients, who had previously undergone surgery elsewhere, consisted of 4 patients who had unsuccesful posterior approach surgeries for central calcified TDH. At our institution, these patients underwent reintervention through an anterior approach with anterior plate and screw placement, resulting in resolution of symptoms upon follow-up.

## 4 Discussion

Thoracic disc herniations are a rare and challenging pathology, requiring careful multidisciplinary management due to their heterogeneity and potential risks associated with surgical treatment. Our study represents one of the largest single-center case series in the literature comparing anterior and posterior approaches for thoracic disc herniation surgery. Both approaches demonstrated notable improvements in clinical outcomes, including Frankel, Nurick, VAS scores and sphincter functionality, with a slightly higher rate of neurological improvement observed in the anterior cohort.

In a 1998 retrospective analysis of 71 patients, Stillerman et al. found no statistically significant difference in neurological outcomes and pain improvement between anterolateral and posterolateral approaches (Stillerman et al., 1998). More recently, Arts et al.compared the mini-transthoracic approach in 56 patients to transpedicular discectomy in 44 patients, reporting satisfactory clinical improvement in both groups regarding neurological outcomes and pain scores (Arts and Bartels, 2014). Further supporting these findings, Hurley et al. conducted a systematic review of 37 studies involving 1156 patients with 1300 TDHs, finding no significant differences in overall neurological improvement or worsening between anterior and posterior approaches (Hurley et al., 2017). Interestingly, the study conducted by Oltulu et al. stands out as the only one reporting better neurological improvement in the anterior group with a rate as high as 85.7 % (Oltulu et al., 2019).

The explanation for these results, as agreed upon also by other authors, primarily hinges on proper surgical indication. The anterior approach is generally preferred for central, giant, and calcified herniations due to its superior visualization, resection capability, and reduced spinal cord manipulation, making it particularly effective for decompressing the ventral spinal canal (Gong et al., 2018; Farber et al., 2024). In our study, 75 % of giant, 77 % of calcified, and 78 % of central herniations were treated with the anterior approach, reflecting this preference. Conversely, posterolateral approaches are favored for paracentral herniations of varying consistency (preferably soft or mixed) and central, non-giant, soft herniations that can be accessed via pediculectomy or costotransversectomy (Lubelski et al., 2013). Indeed, in our subgroup of 4 patients who experienced neurological worsening after posterior/posterolateral surgery at other centers, all had calcified, midline herniations that would have been more appropriately managed with an anterior approach. In fact, these patients subsequently improved neurologically following revision surgery via a transthoracic approach. Our radiological analysis further confirms that, when the approach is correctly chosen, there is no significant difference between anterior and posterior routes in the postoperative area of the spinal canal free from compression.

However, our decision making process introduces a selection bias, as anterior approaches are often reserved for more complex cases, which contributes to higher complication rates and longer operative times. The anterior group had a 20.6 % complication rate compared to 15 % in the posterior group, aligning with broader studies. For instance, an analysis of national hospital discharge data involving over 25,000 patients reported significantly higher complication rates for anterior compared to non-anterior approaches (26.8 % vs. 9.6 %, p < 0.001) (Yoshihara and Yoneoka, 2014). Contrarily, Oltulu et al. reported a higher rate of major complications in the posterior group, however, the anterior group presented an higher overall complications rate due to the higher incidence of minor complications. Respiratory complications were exclusively observed in the anterior group, often due to pleural opening and lung retraction, which could be virtually eliminated with mini-open retropleural or full-endoscopic techniques (Farber et al., 2022; Sofoluke et al., 2024; Silva et al., 2023). The risk of cerebrospinal fluid leakage and pleural fistulas in cases of giant calcified TDHs can be minimized through microsurgical techniques such as the eggshell technique, along with appropriate closure techniques of dural defects (Yang et al., 2016). Other complications, including iatrogenic fractures due to excessive vertebral bone removal, can be mitigated using navigation systems for precise mapping of the drilling zone. Additionally, if more than 50 % of the vertebral body is resected during discectomy, or if there is a pre-existing kyphotic deformity or prior laminectomy without fusion, fixation systems such as anterior plating may be required (Quraishi et al., 2014; Ayhan et al., 2010).

Instead, posterior approaches are often associated with inadequate decompression and/or neurological deterioration. In this regard, it is essential to minimize spinal cord manipulation by employing microsurgical dissection techniques and approaches that allow for a lateral-to-medial working corridor. Moreover, endoscope-assisted surgery can help mitigate these issues by reducing the blind angle ventral to the spinal cord (Baram et al., 2021). Additionally, in our study, 40 % of patients required posterior screw stabilization to address iatrogenic instability resulting from excessive bone removal.

Operative times were longer for the anterior cohort compared to the posterior cohort, consistent with findings from Arts et al. (Arts and Bartels, 2014), that reported longer times for anterior approaches (229 min vs 98 min, p < 0.001). This disparity likely reflects greater surgeon familiarity with posterior anatomy, as well as the necessity for an access surgeon during anterior procedures, which can disrupt flow and extend surgery time compared to the mono-operator posterior surgeries. Our previous study has emphasized the importance of the learning curve in transthoracic TDH surgery, showing progressive reductions in operative time with increased experience (Anania et al., 2021). In line with this, at the beginning of the learning curve, two patients in our case series required reoperation due to insufficient excision of the thoracic herniation as revealed by radiological follow-up. In our opinion, a continuous and voluminous caseload, feasible only in high-volume tertiary spinal surgery centers, is essential for gaining familiarity with the anterior approach and reducing surgical times.

Hospital length of stay was also longer for the anterior group, with a median stay of 11 days compared to 5 days for the posterior group, consistent with Yoshihara et al., that reported a mean stay of 7.6 days for anterior approaches versus 4.8 days for posterior approaches (Yoshihara and Yoneoka, 2014). The more invasive nature of the trans-thoracic transpleural approach, combined with higher complication rates and the need for postoperative monitoring and chest tube management, likely contributes to these results.

Finally, our data did not show differences in outcomes between patients operated on with or without neuromonitoring. Despite mixed literature on its role, some studies suggest that neurophysiological monitoring may be particularly beneficial for complex TDHs, specifically giant or calcified herniations (Hadley et al., 2017; Sala et al., 2018; Cornips et al., 2017; Armocida et al., 2022). Recently, Fehlings et al. recommended intraoperative neurophysiological monitoring for high-risk spine surgery patients, including those with significant cord compression and myelopathy (Fehlings et al., 2024). While our data does not allow to draw conclusions on this matter, in our experience, neuromonitoring has often been valuable in guiding surgical maneuvers and modifying strategies when necessary.

### 4.1 Limitations

This study has several limitations that must be considered. The relatively small sample size of 54 patients limits the statistical power and prevents us from drawing definitive conlcusions regarding the superiority of one approach over the other. The retrospective nature of the study introduces selection bias, as the choice of surgical approach was influenced by herniation characteristics, surgeon preference, and availability of an access surgeon. Given the rarity of TDHs, prospective randomized controlled trials may not be feasible, but larger multicentric datasets could help provide more robust analyses focusing on specific TDH subgroups.

### 4.2 Future directions

The future of TDH surgery is moving towards minimally invasive and endoscopic techniques, aimed at overcoming the limitations of traditional anterior and posterior approaches. While clinical outcomes are now comparable, anterior approaches still provide superior access to central, giant, and calcified herniations. Emerging techniques, such as the mini-open lateral retropleural approach described by Farber et al., have shown promise in reducing the invasiveness of anterior surgeries (Farber et al., 2022). However, the most transformative advance might be the transforaminal endoscopic thoracic discectomy, which avoids pleural and lung manipulation (Konakondla et al., 2022). Silva et al. demonstrated that full-endoscopic discectomy offers low rate of dural tears, recurrent herniation, and myelopathy, while also minimizing soft tissue damage and shortening recovery times (Silva et al., 2023; Bae et al., 2022). This technique has the potential to elevate the safety and efficacy of TDH surgery. Future research should focus on refining these techniques and validating their long-term outcomes through large-scale studies.

## 5 Conclusions

The choice of surgical approach for TDHs should be individualized based on herniation characteristics, surgeon expertise, and patient-specific factors. Anterior approaches are preferred for managing complex central, giant, and calcified herniations, showing higher rates of neurological improvement but with longer operative times, higher complication rates, and extended hospital stays. Posterior approaches are more familiar to spine surgeons and are better suited for paracentral and soft herniations. Although direct comparisons were made between the two approaches, the limited sample size in our study precludes definitive conclusions regarding the superiority of one approach over the other.

## Statements and declarations

Not applicable.

## Ethical considerations

The study received approval from the local ethics committee (approval number 1575473) and adhered to the principles of the 1964 Declaration of Helsinki and its amendments.

## Consent to participate

The necessary patient informed consent was obtained in this study.

## Consent for publication

The necessary patient consent for publication was obtained in this study.

## Data availability

Our dataset cannot be shared due to hospital policy.

## Funding statement

The publication fee for this work was covered by the Italian Ministry of Health's “Ricerca Corrente” funding to the IRCCS Humanitas Research Hospital.

## Declaration of competing interest

The authors declared no potential conflicts of interest with respect to the research, authorship, and/or publication of this article.
